# Glutamine Hydrolysis by Imidazole Glycerol Phosphate Synthase Displays Temperature Dependent Allosteric Activation

**DOI:** 10.3389/fmolb.2018.00004

**Published:** 2018-02-06

**Authors:** George P. Lisi, Allen A. Currier, J. Patrick Loria

**Affiliations:** ^1^Department of Chemistry, Yale University, New Haven, CT, United States; ^2^Department of Molecular Biophysics and Biochemistry, Yale University, New Haven, CT, United States

**Keywords:** allostery, enzyme dynamics, relaxation dispersion, thermophile, NMR

## Abstract

The enzyme imidazole glycerol phosphate synthase (IGPS) is a model for studies of long-range allosteric regulation in enzymes. Binding of the allosteric effector ligand N'-[5'-phosphoribulosyl)formimino]-5-aminoimidazole-4-carboxamide-ribonucleotide (PRFAR) stimulates millisecond (ms) timescale motions in IGPS that enhance its catalytic function. We studied the effect of temperature on these critical conformational motions and the catalytic mechanism of IGPS from the hyperthermophile *Thermatoga maritima* in an effort to understand temperature-dependent allostery. Enzyme kinetic and NMR dynamics measurements show that apo and PRFAR-activated IGPS respond differently to changes in temperature. Multiple-quantum Carr-Purcell-Meiboom-Gill (CPMG) relaxation dispersion experiments performed at 303, 323, and 343 K (30, 50, and 70°C) reveal that millisecond flexibility is enhanced to a higher degree in apo IGPS than in the PRFAR-bound enzyme as the sample temperature is raised. We find that the flexibility of the apo enzyme is nearly identical to that of its PRFAR activated state at 343 K, whereas conformational motions are considerably different between these two forms of the enzyme at room temperature. Arrhenius analyses of these flexible sites show a varied range of activation energies that loosely correlate to allosteric communities identified by computational methods and reflect local changes in dynamics that may facilitate conformational sampling of the active conformation. In addition, kinetic assays indicate that allosteric activation by PRFAR decreases to 65-fold at 343 K, compared to 4,200-fold at 303 K, which mirrors the decreased effect of PRFAR on ms motions relative to the unactivated enzyme. These studies indicate that at the growth temperature of *T. maritima*, PFRAR is a weaker allosteric activator than it is at room temperature and illustrate that the allosteric mechanism of IGPS is temperature dependent.

## Introduction

**Figure 1:**
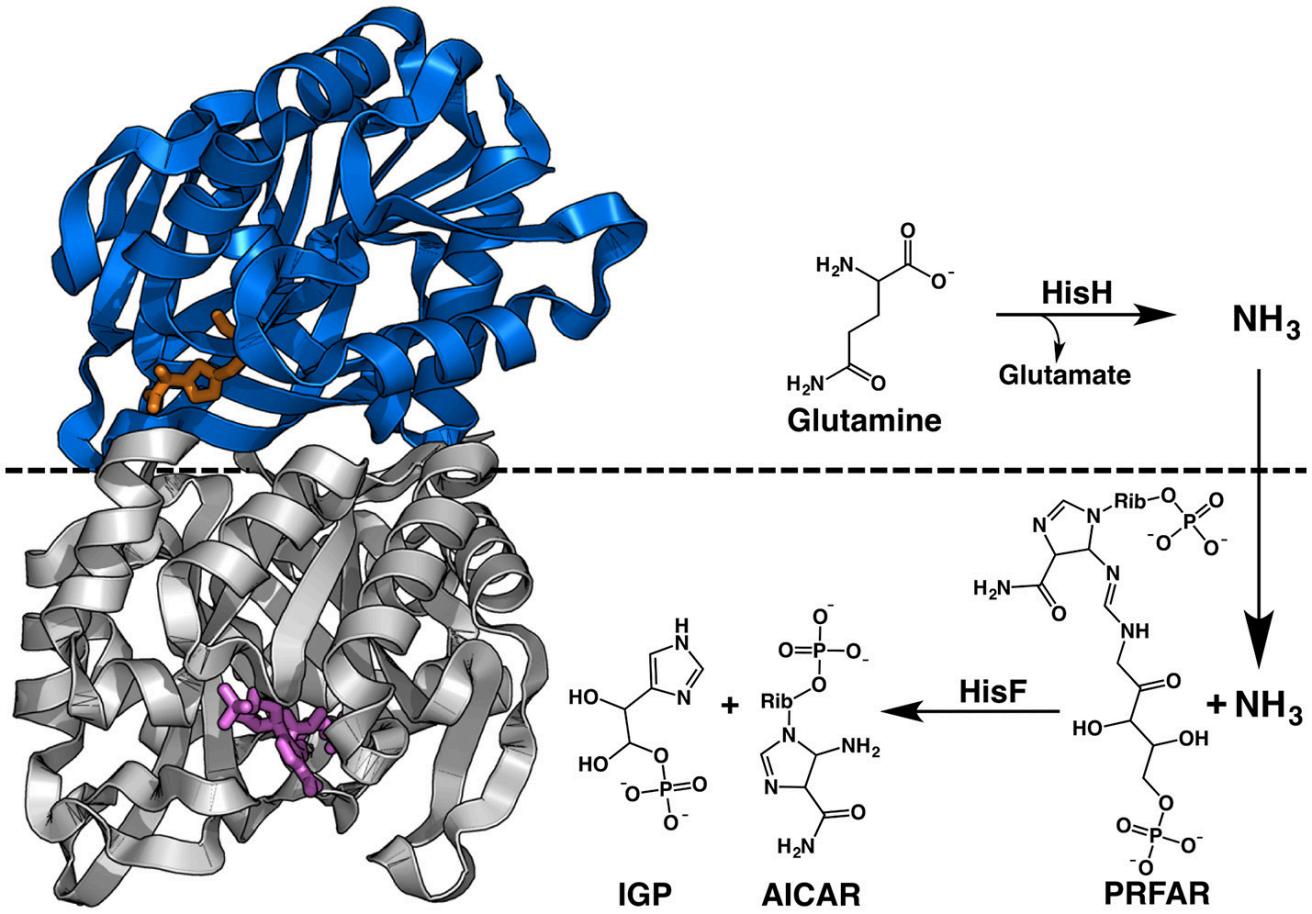
Structure and reaction of *T. maritima* IGPS. This HisH subunit, in blue, contains the glutaminase site with the Gln analog acivicin shown in orange sticks. The HisF subunit is colored gray with PRFAR shown in purple sticks. The non-covalent interface of the dimeric complex is highlighted by the dashed black line, which also denotes the subunit responsible for each portion of the chemical reaction.

Allostery is a ubiquitous biological regulatory mechanism that is essential for cell growth and adaptation. However, the molecular details by which information is transmitted between effector sites and active sites in enzymes is an unresolved area of research. Although the intricate balance between biomolecular stability and flexibility has been studied for decades (Fersht et al., 1991; Fersht and Serrano, 1993; Hollien and Marqusee, 1999, 2002; Miller et al., 2002; Robic et al., 2002; Ratcliff et al., 2009; Mallamace et al., 2016; McClelland and Bowler, 2016), it is unclear to what extent the structural composition of a protein or makeup of its chemical environment drive allostery. Many recent investigations of allostery have concluded that protein dynamics can be as important as secondary, tertiary, or quaternary structures in directing these biochemical mechanisms (Popovych et al., 2006; Petit et al., 2009; Bu and Callaway, 2011; Motlagh et al., 2012, 2014; Manley et al., 2013; Choi et al., 2015). These and other reports have established dynamic allostery as a widespread biological phenomenon, but one of the facets of these mechanisms that has remained unexplored is the effect of temperature. Temperature and structural motion are certainly connected, and theories about the temperature dependence of enzyme-catalyzed rates have been resurgent as of late (Bera et al., 2000; Onuchic et al., 2006; Arcus et al., 2016; Doyle et al., 2016; Katava et al., 2016; Roy et al., 2017). For enzymes reliant on flexibility for allosteric function, temperature is likely to play a significant role in governing these mechanisms. To gain insight into the effect of temperature on allosteric communication, we examined molecular motions and catalytic activity in imidazole glycerol phosphate synthase (IGPS) from the hyperthermophilic bacterium *Thermatoga maritima*, which has an optimal growth temperature of ~ 353 K and displays V-type allostery (altered catalytic V_max_, as opposed to K-type allostery, affecting ligand affinity; Monod et al., 1965). IGPS functions at the branch point of the histidine and purine biosynthetic pathways in bacteria, archea, and plants, catalyzing glutamine (Gln) hydrolysis in its HisH subunit and the cyclization of N'-[5′-phosphoribulosyl)formimino]-5-aminoimidazole-4-carboxamide-ribonucleotide (PRFAR) in its HisF domain (Figure 1), which form a nanomolar affinity complex (Lipchock, 2010). Previous investigations of IGPS, carried out at 298–303 K (near ambient temperature), highlighted a strong dependence of Gln hydrolysis on the presence of allosteric effectors such as PRFAR, which activates IGPS glutaminase catalysis 4,900-fold over basal levels (Klem and Davisson, 1993; Beismann-Driemeyer and Sterner, 2001; Myers et al., 2005; Lisi et al., 2016), and demonstrated that IGPS utilizes extensive millisecond (ms) timescale flexibility throughout its effector binding domain, HisF, for catalytic function (Lipchock and Loria, 2010; Rivalta et al., 2012; Lisi et al., 2016, 2017). Computational community network analyses also revealed that local regions of the HisF and HisH domains undergo correlated motion in order to propagate allosteric signals and that dynamic crosstalk between these small communities is altered in the presence of PRFAR (Amaro et al., 2007; Rivalta et al., 2012). Here, we sought to exploit the thermostability of this allosteric enzyme to further investigate the relationship between temperature, conformational motions, and its allosteric activation.

## Materials and methods

Analytical grade chemicals and antibiotics for protein expression were purchased from AmericanBio (Natick, MA). 3-acetylpyridine adenine dinucleotide (APAD) and glutamate dehydrogenase (GDH) used in kinetic assays were purchased from Santa Cruz Biotechnology (Dallas, TX) and Affymetrix (Santa Clara, CA), respectively, and PRFAR was synthesized as previously described (Lipchock and Loria, 2010).

### Protein expression and purification

The HisH, containing a C-terminal histidine affinity tag, and HisF plasmids were transformed into BL21(DE3) cells as previously described (Lipchock and Loria, 2010). The HisH and HisF proteins were expressed separately at 310 K in M9 minimal medium containing CaCl_2_, MgSO_4_, and MEM vitamins. HisF was grown in 1.5 L of deuterated M9 supplemented with ^15^NH_4_Cl (Cambridge Isotope Labs, Tewksbury, MA) and ^12^C_6_H_12_O_6_ as the sole nitrogen and carbon sources, respectively. Isotopic labeling of isoleucine, leucine, and valine (ILV) methyl groups in HisF was achieved with 60 mg/L of alpha-ketobutyric acid [methyl-^13^C; 3,3-D_2_] and 100 mg/L of alpha-ketoisovaleric acid [3-methyl-^13^C; 3,4,4,4-D_4_] (Cambridge Isotope Labs) added 30 min prior to induction (Tugarinov and Kay, 2003; Tugarinov et al., 2006). HisH was grown in 1 L of deuterated M9 with naturally abundant nitrogen and carbon isotopes. Cultures of both subunits were grown to an OD_600_ of 0.8–1.0 before induction with 1 mM IPTG.

Cells were incubated an additional 7 h at 310 K and harvested by centrifugation. HisF and HisH cell pellets were resuspended in 10 mM Tris, 10 mM CAPS, 300 mM NaCl, and 1 mM β-mercaptoethanol at pH 7.5 and the resulting suspensions were mixed and co-lysed by ultrasonication. The lysis mixture also contained 1 mM phenylmethylsulfonyl fluoride (PMSF). Cell debris was removed by centrifugation and the supernatant was incubated at 333 K for ~30 min to remove unwanted proteins and subsequently mixed with Ni-NTA agarose resin equilibrated with 10 mM Tris, 10 mM CAPS, 300 mM NaCl, and 1 mM β-mercaptoethanol at pH 7.5 for 15–20 min. The resin mixture was added to a gravity column and washed with ~100 mL of the same buffer. The column was then washed with ~100 mL of the same buffer with 15 mM imidazole at pH 9.5. The IGPS complex was eluted with an identical buffer containing 250 mM imidazole at pH 9.5. The eluent was exhaustively dialyzed against 10 mM HEPES, 10 mM KCl, and 0.5 mM EDTA at pH 7.3. Following dialysis, the sample was concentrated and transferred to an identical buffer containing 5% D_2_O using an Amicon centrifugal cell (EMD Millipore, Billerica, MA).

### Ligand titrations and high temperature NMR

NMR titrations were performed on a Varian Inova 600 MHz spectrometer by collecting a series of ^1^H-^15^N TROSY heteronuclear single quantum coherence (HSQC) spectra with increasing PRFAR concentration. Experiments were performed at 303 K and the ^1^H and ^15^N carrier frequencies were the water resonance and 120 ppm, respectively. IGPS was titrated with PRFAR until no further chemical shift perturbations were detected, to a final ligand concentration of 1.0 mM.

NMR samples used at high temperatures were prepared with a 0.5 cm layer of mineral oil atop the protein solution to prevent reflux of the aqueous phase. Prior to data collection, samples were “degassed” in NMR tubes by incubation in a heat block at 338 K for at least 1 h. Multiple quantum (MQ) Carr-Purcell-Meiboom-Gill (CPMG) experiments probing ILV methyl group (^13^CH_3_) dynamics were performed on Varian Inova 600 MHz and Agilent 800 MHz spectrometers at 303, 323, and 343 K in a manner described previously (Korzhnev et al., 2004a,b). A constant relaxation period of 0.03 s, a 2.0 s recycle delay, and a τ_*cp*_ array of 0.0, 0.4412, 0.46875, 0.50, 0.68182, 0.75, 1.07143, 1.5, 1.875, 2.5, 3.75, and 7.5 ms were used in the CPMG pulse sequence. NMR spectra were processed with NMRPipe (Delaglio et al., 1995) and analyzed in SPARKY (Goddard and Kneller, 2008). Transverse relaxation rates (R_2_) were determined from peak intensities of the resonances using in-house scripts. Relaxation dispersion curves at two static magnetic fields were generated from fits to the fast-limit CPMG equation by plotting R_2_ vs. 1/τ_*cp*_ in GraphPad Prism 7.0 (GraphPad Software), where uncertainty values were obtained from replicate spectra. Dual field relaxation dispersion data were also analyzed using RELAX (d'Auvergne and Gooley, 2008a,b; Bieri et al., 2011) with the R2eff, NoRex, and MMQCR72 (two-site Carver-Richards) models (Morin et al., 2014).

For resolved resonances in methyl CPMG relaxation dispersion experiments with sufficient signal-to-noise at 303, 323, and 343 K, the apparent activation barrier (*E*_*a*_) for conformational exchange (*k*_*ex*_) was determined by fits to the Arrhenius expression (Equation 1). In cases where data were available at two temperatures, *E*_*a*_ was determined using the integrated form of the Arrhenius expression (Equation 2).

(1) $$k_{ex}=A\times e^{-E_{a}/RT}$$

(2) $$\ln\left(\frac{k_{ex,1}}{k_{ex,2}}\right)=\frac{E_{a}}{R}\left(\frac{1}{T_{1}}-\frac{1}{T_{2}}\right)$$

### Enzyme kinetics

IGPS glutaminase activity was measured in presence and absence of PRFAR by adapting the procedure described by Klem and Davisson (1993). Stock solutions of IGPS (0.1 mM), PRFAR (120 mM), and glutamine (48 mM) were prepared in 50 mM Tris-HCl, 50 mM KCl, and 1 mM EDTA at pH 8.0. Apo IGPS ([IGPS] ~ 1 μM) and PRFAR-bound IGPS ([PRFAR] ~ 5 mM) were incubated with 0–15 mM glutamine at 303 K for 20 min. The reactions were quenched by immediate boiling of the solutions for 4 min followed by freezing. Identical assays were carried out at temperatures of 313, 323, 333, 343, and 353 K with the incubation time varying between 5 and 20 min, depending on the window of linearity for initial velocity profiles.

Subsequently, glutamate production was quantified with a coupling reaction utilizing GDH and APAD. Stock solutions of GDH (10 mg/mL) and APAD (70 mM) were made in 50 mM Tris-HCl, 50 mM KCl, 1 mM EDTA at pH 8.0, and added to final concentrations of 100 μg (GDH) and 0.5 mM (APAD) to aliquots of the previously frozen reactions. The thawed reaction mixtures were incubated at 310 K for 60 min after which the concentration of APADH produced during the GDH catalyzed conversion of glutamate to 2-oxoglutarate was determined at 363 nm (ε_363_ = 8,900 M^−1^ cm^−1^) using a Cary 100 UV-Visible spectrophotometer (Agilent Technologies). Kinetic data were analyzed with the Michaelis–Menten kinetic model in GraphPad Prism 7.0.

Determination of thermodynamic parameters from enzyme kinetic experiments was carried out as follows. The free energy (Δ*G*^†^), enthalpy (Δ*H*^†^), and entropy (Δ*S*^†^) of activation for PRFAR-stimulated Gln hydrolysis were determined from the Eyring and Arrhenius expressions (Equations 3 and 4) and from Equation (5).

(3) $$\ln k_{cat}=\ln\left(\frac{k_{b}T}{h}\right)-\Delta G^{†}/RT$$

(4) $$\Delta H^{†}=E_{a}-RT$$

(5) $$T\Delta S^{†}=\Delta H^{†}-\Delta G^{†}$$

In these equations *k*_*b*_, *h*, and *R* are Boltzmann's, Planck's, and the gas constants, respectively, where *T* is the temperature and *E*_*a*_ is the activation energy. For basal Gln hydrolysis, the kinetic data were modeled as shown in Equation (6),

(6) $$\begin{array}{l} \ln k=\ln\left(\frac{k_{b}T}{h}\right)\times \left(\frac{\Delta H^{†}+\Delta C_{p}\left(T-T_{0}\right)}{RT}\right)\\ \;+\;\left(\frac{\Delta S^{†}+\Delta C_{p}\times \ln\left(\frac{T}{T_{0}}\right)}{R}\right) \end{array}$$

where Δ*C*_*p*_ is the change in heat capacity and *T*_0_ is the reference temperature, in this case 303.15 K. The apparent coupling free energy, *Q*_*ax*_, was determined from Equation (7) to reflect the energetic magnitude of the allosteric effect asserted by PRFAR on IGPS (Tlapak-Simmons and Reinhart, 1998; Carlson and Fenton, 2016).

(7) $$Q_{ax}=\frac{K_{m}^{0}}{K_{m}^{\infty }}$$

In Equation (7), the apparent coupling free energy *Q*_*ax*_ equals the ratio of the *K*_*m*_ for Gln in the absence ($K_{m}^{0}$) and presence of saturating PRFAR ($K_{m}^{\infty }$). The coupling entropy and enthalpy were obtained through the following relationship.

(8) $$\ln Q_{ax}=\frac{\Delta S_{ax}}{R}-\frac{\Delta H_{ax}}{R}\left(\frac{1}{T}\right)$$

## Results

### Enzyme kinetics

To examine the temperature dependence of IGPS allostery, the glutamine hydrolysis reaction was monitored in the absence (basal) and presence (activated) of saturating PRFAR from 303 to 353 K. Several control experiments were performed to ensure the stability of IGPS, PRFAR, and Gln at elevated temperatures. First, IGPS was incubated at 343 K for 20 min (5-fold longer than the kinetics assay) and its activity was measured at 303 K. No difference in kinetic parameters were observed relative to IGPS that had been incubated only at 303 K, indicating that IGPS is stable at and does not lose activity after incubation at 343 K, consistent with its temperature independent circular dichroism (CD) spectra (Figure S1). Likewise, Gln and PRFAR were incubated at 343 K for 2 h and their ^1^H NMR spectra were compared to those obtained before incubation (Figure S2). No additional resonances appeared after high temperature incubation and peak intensities were within 10% of their pre-incubation value. To ensure that PRFAR was binding to IGPS at the elevated temperatures, an experiment was performed at 343 K to measure the *K*_*act*_ for PRFAR (the concentration of PRFAR that gives 50% enhancement of Gln hydrolysis) in which [PRFAR] is varied at saturating [Gln]. Figure S3 shows that the PRFAR *K*_*act*_ is 6.6 μM at 343 K, which is very close to its room temperature *K*_*d*_-value of 1–3 μM determined by isothermal titration calorimetry (Lipchock and Loria, 2010; Lisi et al., 2016). This result indicates that PRFAR was present at saturating concentrations during all kinetic assays and that its affinity is largely temperature independent (Figure S3). Thus, in the temperature range and time course of the reaction, there appears to be no significant degradation of enzyme, substrate, or allosteric effector.

**Figure 2:**
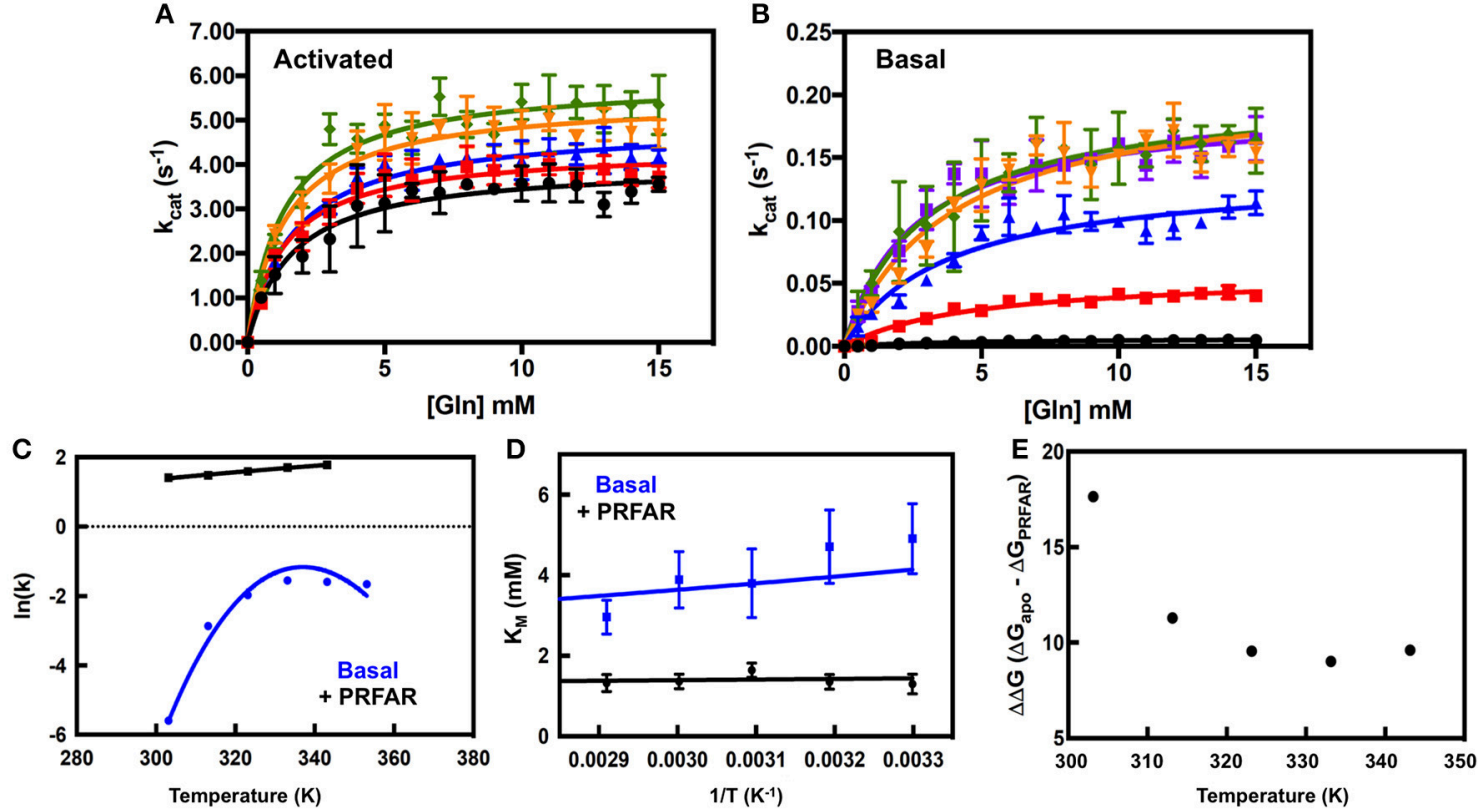
Analysis of IGPS catalytic activity. Glutaminase profiles in the presence **(A)** and absence **(B)** of PRFAR were fit for Michaelis–Menten kinetic parameters and error bars are based on *n* ≥ 3 measurements and in some cases are smaller than the size of the data point. Traces are color coded as follows: black (303 K), red (313 K), blue (323 K), orange (333 K), green (343 K), and purple (353 K, Basal). Corresponding Arrhenius plots from kinetic traces of activated and basal IGPS are shown in **(C)** and the temperature dependence of *K*_*m*_ is shown in **(D)**. The temperature driven changes in free energy (ΔΔ*G*) between apo and PRFAR-bound IGPS are shown in **(E)** as a difference of (Δ*G*)_*apo*_ – (Δ*G*)_*PRFAR*_ obtained using the Eyring expression.

Kinetic traces monitoring glutamate production catalyzed by IGPS are shown in Figure 2. The measured Gln *K*_*m*_-values are, just as PRFAR binding, relatively insensitive to temperature (Table 1). The Gln *K*_*m*_ in the presence of PRFAR is invariant over the temperature range whereas for basal Gln binding, *K*_*m*_ decreases by 1.6-fold between 303 and 353 K. This leads to van't Hoff plots yielding Δ*G* = – 0.9 ± 0.1 and Δ*G* = −3.6 ± 0.2 kJ/mol for steady-state Gln binding in the presence and absence of PRFAR, respectively (Figure 2). This study also reveals that in addition to PRFAR being a V-type activator, it is also a weak K-type activator as indicated by the slight decrease in Gln *K*_*m*_ in the presence of PRFAR, relative to that of the basal reaction. The apparent coupling free energy, *Q*_*ax*_, obtained from this analysis is −2.7 ± 0.2 kJ/mol (Figure 2).

**Table 1:** Kinetic parameters determined from glutaminase assays as a function of temperature.

**Temp (K)**	**Activated**			**Basal**			**(*k_*cat*_*/*K_*m*_*)^act^/ (*k_*cat*_*/*K_*m*_*)^basal^**
	***k_*cat*_* (s^−1^)**	***K_*m*_* (mM)**	***k_*cat*_*/*K_*m*_***	***k_*cat*_*(s^−1^)**	***K_*m*_* (mM)**	***k_*cat*_* /*K_*m*_***	
303.15	4.09 ± 0.13	1.30 ± 0.25	3.15 × 10^3^	3.72 (0.04) × 10^−3^	4.91 ± 0.87	7.58 × 10^−1^	4,161
313.15	4.37 ± 0.12	1.35 ± 0.18	3.24 × 10^3^	5.71 (0.04) × 10^−2^	4.71 ± 0.91	1.21 × 10^1^	268
323.15	4.89 ± 0.11	1.64 ± 0.18	2.98 × 10^3^	1.39 (0.01) × 10^−1^	3.80 ± 0.85	3.65 × 10^1^	82
333.15	5.50 ± 0.15	1.36 ± 0.18	4.04 × 10^3^	2.12 (0.01) × 10^−1^	3.89 ± 0.70	5.44 × 10^1^	74
343.15	5.92 ± 0.18	1.32 ± 0.21	4.48 × 10^3^	2.04 (0.08) × 10^−1^	2.96 ± 0.42	6.88 × 10^1^	65

*Michaelis–Menten parameters were determined in the presence (activated) and absence (basal) of saturating PRFAR*.

An interesting observation is the rather modest (2-fold) increase in *k*_*cat*_ for PRFAR activated glutamine hydrolysis (Figure 2A) between 303 and 343 K, yielding an Eyring activation free energy of 8.4 ± 0.5 kJ/mol (Figure 2C). In contrast, temperature has a much more significant effect on *k*_*cat*_ of the basal Gln hydrolysis reaction (Table 1, Figure 2B). This differential temperature effect leads to diminished activation by PRFAR over basal catalysis, which is only 65-fold at 343 K, a significant attenuation of its allosteric effect that is >4,000-fold at 303 K (Figure 2E). Unlike the linear Eyring plots for *k*_*cat*_ in the presence of PRFAR, apo IGPS displays concave Eyring profiles with catalytic activity actually decreasing slightly at the highest temperature (Figure 2D). As noted previously this activity decrease does not appear to be due to protein or substrate degradation. The temperature dependence of *k*_*cat*_ for the basal Gln hydrolysis reaction is modeled assuming a temperature dependence of activation enthalpy (Δ*H*^†^) and entropy (Δ*S*^†^) due to a difference in heat capacity between the ground and transition states (Hobbs et al., 2013).

### NMR studies

Prior work on IGPS demonstrated a close connection between ms motions and the allosteric activation of Gln hydrolysis (Lipchock and Loria, 2010; Rivalta et al., 2012). It was shown that PRFAR binding enabled concerted ms motions throughout the HisF domain that were important for maximal catalytic activity. We demonstrated, using a library of allosteric effectors, that the ability of the ligand to enhance Gln hydrolysis rates was directly correlated to its ability to induce these ms motions (Lisi et al., 2016; Rivalta et al., 2016). Moreover, mutation of single, critical residues in HisF resulted in attenuation of PRFAR-induced motions and a corresponding decrease in Gln hydrolysis rates in the presence of PRFAR, but not in its absence (Lisi et al., 2017). Given these established links between ms motions and allostery, we investigated the effect of temperature on IGPS flexibility by solution NMR spectroscopy.

**Figure 3:**
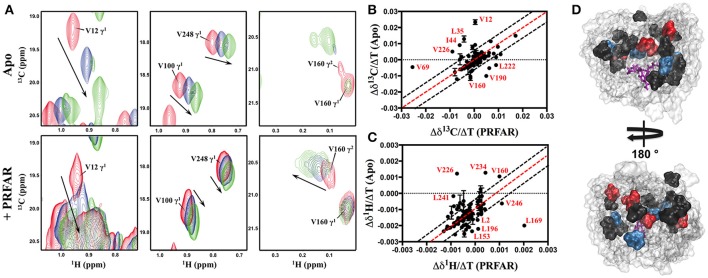
**(A)** Representative NMR spectral overlays of HisF ^13^CH_3_-ILV methyl groups in apo (upper) and PRFAR-bound IGPS (lower) showing temperature-dependent resonance shifts. Resonances in red correspond to spectra collected at 303 K, blue to 323 K, and green to 343 K while arrows indicate the direction of shifts with increasing temperature. Correlations between the temperature dependencies of chemical shifts in ^1^H^13^CH_3_-ILV spectra of apo and PRFAR-bound IGPS are shown for the carbon **(B)** and proton **(C)** dimensions. Temperature-dependent shifts outside of 90% confidence boundaries are mapped onto the HisF structure in **(D)**, where areas in black denote residues with proton shifts outside of these boundaries, red denotes carbon shifts outside of these boundaries, and blue denotes residues with both proton and carbon shifts outside of these boundaries.

NMR spectra of apo IGPS (^2^H,^13^CH_3_-ILV,^15^N HisF; ^2^H HisH) demonstrate significant thermostability, clearly resolving most HisF ^13^CH_3_-ILV resonances over a temperature range of 303–343 K (Figures S4, S5). IGPS is stable at 343 K for ≥80 h, as controls utilizing two-dimensional ^1^H^15^N and ^13^CH_3_-ILV spectra of PRFAR-saturated IGPS collected before and after long relaxation experiments display nearly identical features. Further, it is clear from these data that HisF and HisH remain complexed at each of the studied temperatures, as the measured R_2_-values are consistent with a dimeric IGPS structure and are significantly higher than those of the HisF monomer in solution. The majority of ILV resonances in apo IGPS undergo linear, temperature-dependent shifts, however, at the highest temperature, some methyl resonances experience slow chemical exchange behavior (V12, L35, I129, I151, V160, I198). Temperature dependent chemical shift trajectories in PRFAR-bound IGPS are also generally linear (Figure 3), however instances of slow exchange at 343 K are prevalent in five resonances, corresponding to I42, V66, L152, V160, and I232. We compared the slope of chemical shift changes with temperature (δ^13^C/ΔT and δ^1^H/ΔT) in apo and PRFAR-bound IGPS and in general, most of the chemical shifts respond similarly to temperature complexes (Figure 3, Figure S6). Interestingly, the majority of residues that deviate from this observation in either the carbon or proton dimensions are those with previously identified ms conformational motions (Lipchock and Loria, 2010; Lisi et al., 2016, 2017), illustrating some significant differences in the temperature-dependent flexibility between the apo and PRFAR-activated IGPS.

**Figure 4:**
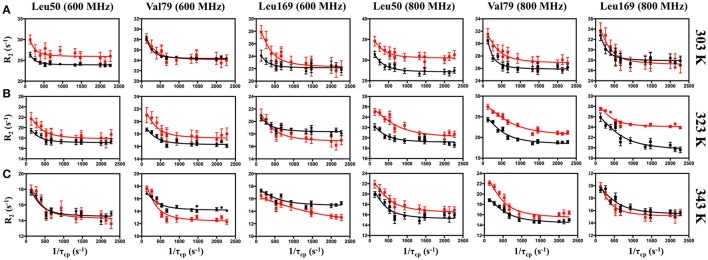
Representative CPMG curves collected at 600 (left panels) and 800 MHz (right panels) on apo (black) and PRFAR-bound (red) IGPS at **(A)** 303 K, **(B)** 323 K, and **(C)** 343 K. Methyl group assignments are indicated at the top of each column. Error bars were determined from duplicate experiments.

For more quantitative characterization, we examined the temperature dependence of millisecond motions, utilizing ^13^CH_3_-ILV MQ CPMG relaxation dispersion experiments (Ollerenshaw et al., 2003). Representative dispersion curves for apo IGPS are shown in Figure 4 for experiments carried out at 303, 323, and 343 K. These methyl groups represent some of the well-established sites of flexibility within the HisF domain, namely those near the PRFAR binding site and hydrophobic Leu47-Val48-Phe49-Leu50 cluster. Complete summaries of relaxation dispersion results are provided in Tables S1–S4.

**Figure 5:**
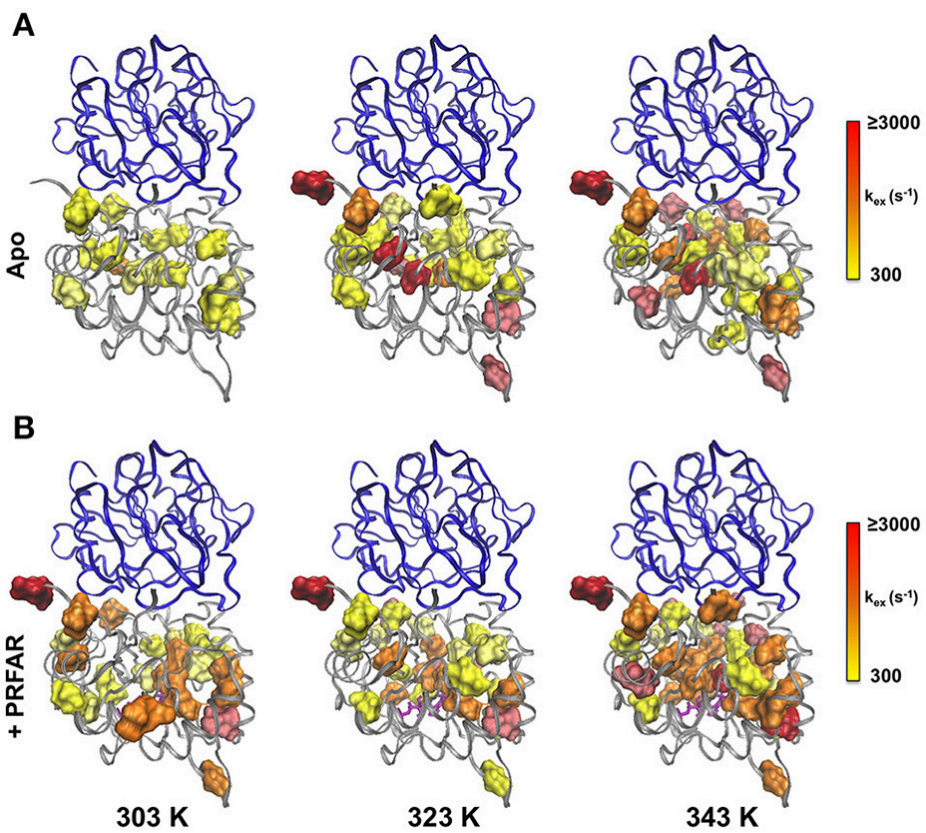
Clustering of *k*_*ex*_-values determined from CPMG relaxation dispersion experiments on apo (upper panel) and PRFAR-bound IGPS (lower panel). The distributions of *k*_*ex*_-values are shown for experiments carried out at 303, 323, and 343 K according to the inset scales, where optimal bin sizing was determined using a procedure outlined by Scott (1979).

In apo IGPS at 303 K, a total of 20 ILV methyl group resonances (out of 116 possible) exhibit millisecond motions, consistent with previous reports that IGPS is relatively inflexible in its apo form (Lipchock and Loria, 2010; Lisi et al., 2016). As the sample temperatures approach the native growth environment of *T. maritima* (~ 353 K) additional methyl containing amino acids exhibit measureable relaxation dispersion curves. At 343 K, 42 methyl groups in the apo enzyme undergo ms motion (Table S1). In the presence of PRFAR (Figure S3), flexibility in the form of measurable dispersion curves is observed in 29 and 37 ILV methyl groups at 303 and 323 K, respectively (Figure 4). However, at 343 K, relaxation dispersion experiments reveal 43 flexible residues, the same number observed for apo IGPS at this temperature. The ranges of *k*_*ex*_-values determined from relaxation dispersion experiments show that the kinetics of methyl group motions are clustered between 300–1,000 and 250–1,500 s^−1^ in apo and PRFAR-bound IGPS at 303 K, respectively. Increasing temperatures shift these ranges to 500–2,500 s^−1^ at 323 K and 600–2,500 s^−1^ at 343 K, however, the distributions of *k*_*ex*_-values among dynamic methyl groups are quite different for apo and effector-bound enzymes (Figure 5, Figure S7).

**Figure 6:**
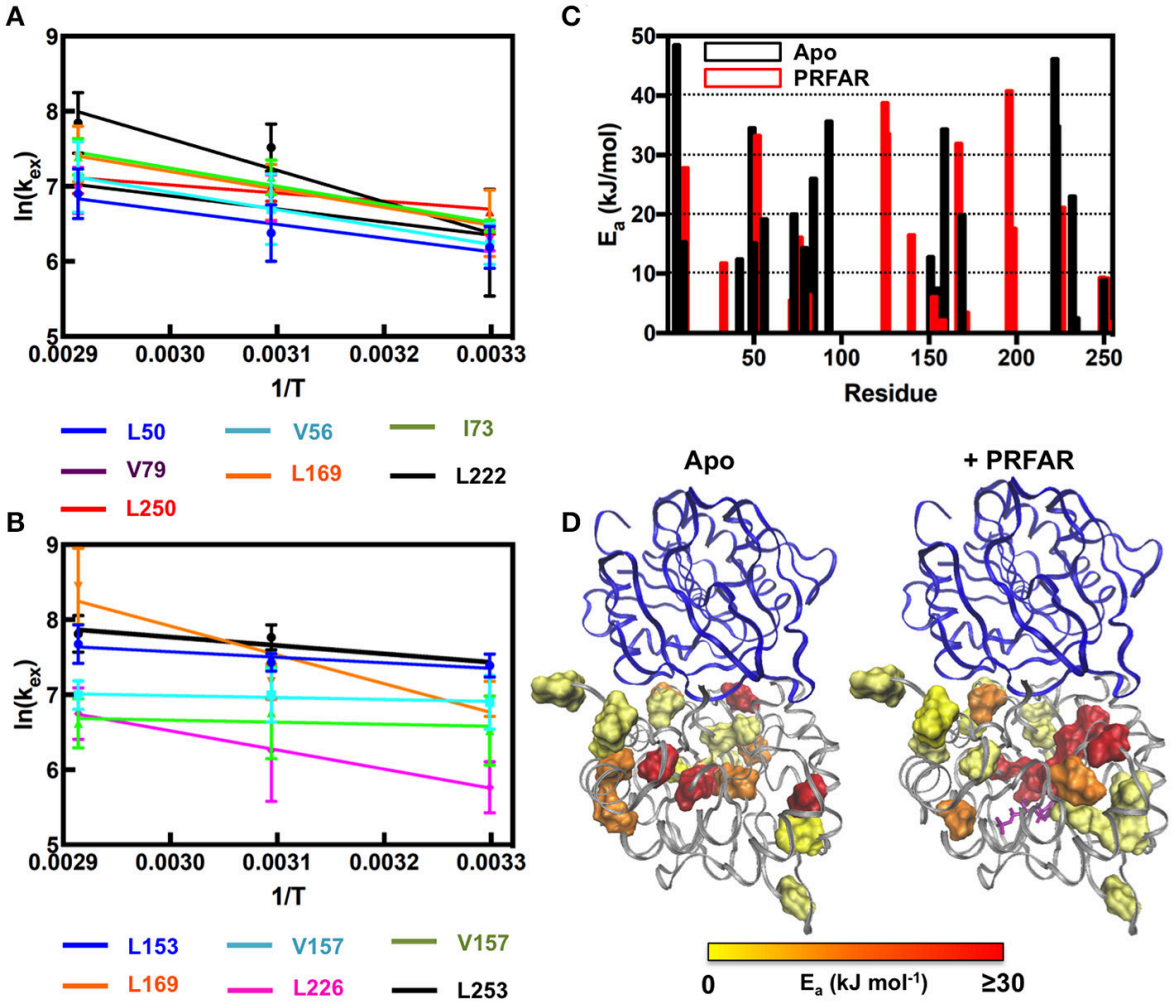
Arrhenius plots for **(A)** apo and **(B)** PRFAR-bound IGPS based on temperature-dependent *k*_*ex*_-values determined from NMR relaxation dispersion experiments. *k*_*ex*_-values were determined from simultaneous fitting of single residue relaxation data obtained at 800 and 600 MHz. A summary of activation energies determined from fitting NMR relaxation data to the Arrhenius equation (data at three temperatures) or the integrated form of the Arrhenius equation (data at two temperatures) is shown in **(C)** and the resulting values are mapped onto the IGPS structure in **(D)**.

Using these temperature dependent relaxation dispersion results, we determined the activation barrier for conformational motion in several apo (L50, V56, I73, V79, L169, L222, and L250) and PRFAR-bound (L153, V157, V157, L169, L226, L253) IGPS methyl groups that exhibited measurable relaxation dispersion curves at each of the three temperatures studied. For these residues, the energies of activation were determined by non-linear fitting with the Arrhenius expression. Many other resonances in apo (I6, L10, V12, V100, L153, V157, L169, L222, and I232) and PRFAR-bound (V18, I42, L50, I73, V79, I83, V134, V134, and L170) IGPS exhibited reliable dispersion curves at only two temperatures. In these cases, the energies of activation were determined using the integrated form of the Arrhenius equation. The temperature dependence of *k*_*ex*_ is stronger for apo IGPS than it is for the PRFAR-activated enzyme, and activation barriers for motion are non-uniform in the apo enzyme, ranging from 9 to 35 kJ/mol. Non-uniformity in activation barriers is also observed in PRFAR-bound IGPS, ranging from 2 to 31 kJ/mol, however, the PRFAR-bound activation barriers are generally lower than those of the apo enzyme (Figure 6).

## Discussion

IGPS from the hyperthermophile *T. maritima* is a model enzyme for studies of dynamic allostery, and our previous work has detailed many of the important factors for allosteric activation of glutaminase chemistry. Binding of the glutamine substrate or substrate analog acivicin (Chittur et al., 2001) has a small thermodynamic impact on IGPS, and millisecond motions are not altered above the basal level (Lisi et al., 2016). However, interactions of the enzyme with several allosteric effectors, most notably PRFAR, show a favorable entropic driving force and stimulate millisecond flexibility to significantly enhance catalytic rates (Lipchock and Loria, 2010; Lisi et al., 2016). In addition, prior work has demonstrated a close connection between the ability of the allosteric effector to enhance ms motions and its ability to accelerate catalysis above the basal level. Computational and NMR studies have shown that these ms motions are largely confined to the HisF subunit, yet they propagate across the HisF/HisH interface and enhance flexibility of the oxyanion strand in HisH. These enhanced motions enable conformational sampling of a favorable oxyanion hole, thereby facilitating catalysis. These previous experiments were performed near room temperature; the thermophilic nature of *T. maritima* warrants that these experiments be extended to higher temperatures near the growth conditions of this organism.

First, we addressed the temperature dependence of IGPS hydrolysis of glutamine without (basal) and with (activated) PRFAR bound. It is well-known that temperature influences catalytic rates of enzymes (Laidler and Peterman, 1979; Daniel and Cornish-Bowden, 2013), although the interplay between structure and dynamics and their role in temperature-dependent catalysis has not been fully clarified. It is also unclear how enzyme motions, particularly those that are closely tied to allosteric and/or catalytic function, depend on temperature and what implications alterations of these dynamics have for protein allostery (Braxton et al., 1994; Kimmel and Reinhart, 2000; Reinhart, 2004). Several discussions have noted attenuations of flexibility in thermophilic enzymes below their physiological temperatures, however these observations do not appear to be generally applicable to all enzymes (Kohen and Klinman, 2000; Akke, 2004; Liang et al., 2004; Oyeyemi et al., 2010). Previous NMR investigations have detailed structural and dynamic changes in proteins over large temperature ranges (Morino et al., 1984; Jung et al., 2004; Weininger et al., 2014), while others have used temperature to explore origins of evolutionary divergence in thermophilic and mesophilic enzyme pairs (Butterwick et al., 2004; Wolf-Watz et al., 2004; Butterwick and Palmer, 2006; Toth et al., 2009; Daily et al., 2011). Interestingly, a recent study by Reinhart and coworkers demonstrated that fructose-6-phosphate binding to the thermophilic allosteric enzyme phosphofructokinase (PFK) was entropically driven, whereas the same process in a corresponding *Escherichia coli* PFK was not, showing that even in homologous enzymes, allostery may have distinct mechanistic features (McGresham et al., 2014).

Thermophilic IGPS is an enzyme in which part of its allosteric mechanism involves effector-induced ms motion. Motion stimulated at the source of allosteric ligand binding has been shown to propagate to the HisH glutaminase site, specifically the Pro49-Gly50-Val51-G52 loop, consistent with computational studies (Lipchock and Loria, 2010; Rivalta et al., 2012; Lisi et al., 2016). The overall degree of flexibility in the effector binding domain (HisF) is related to catalytic activation, and the extent that global motion is induced throughout the entirety of HisH is under investigation in other studies. *T. maritima* IGPS represents an excellent target to investigate the energetics and dynamics involved in allostery, given that it is active and stable over a very large temperature range. All prior experimental studies with IGPS have taken place at room temperature (or slightly higher, i.e., 303 K). Under these laboratory conditions, IGPS is 50°-60° below the temperature at which it has evolved to function. Whether this close correlation of allosteric activation and ms motions remained at elevated temperatures was an objective of this current study. When the enzymatic function of IGPS is examined between 303 and 353 K, several interesting features emerge. First, in the presence of the allosteric activator PRFAR, the activation barrier for Gln hydrolysis has weak temperature dependence, increasing by only 2-fold over a 50° range (Figure 2). Such a small free energy of activation (8 kJ/mol) is more characteristic of psychrophilic enzymes where the low activation barrier leads to higher *k*_*cat*_-values at their lower growth temperatures (Low et al., 1973; Low and Somero, 1974, 1976; Lonhienne et al., 2000). This suggests that in the presence of PRFAR, the changes in the enzyme structure, which enable hydrolysis of Gln, mainly involves the formation and breakage of weak chemical bonds. In contrast, the basal rate of Gln hydrolysis increases 55-fold over this same temperature range indicating a significantly higher energy barrier to hydrolysis. Between 303 and 313 K the rate of Gln hydrolysis increases 15-fold, corresponding to an enthalpic barrier of 200 kJ/mol. Thus, in the absence of PRFAR, Gln hydrolysis requires more extensive bond rearrangements in IGPS for catalysis to occur, perhaps offering an explanation for the negligible basal activity of IGPS at 298 K.

Temperature-dependent enzyme catalysis is generally theorized to yield exponential activity increases followed by decreases due to a loss of active protein concentration with thermal unfolding or inactivation (Daniel and Danson, 2010). However, numerous examples, including the findings reported herein, show catalytic activities lower than standard models would predict at high temperatures (Thomas and Scopes, 1998; Arnott et al., 2000; Daniel et al., 2001; Eisenthal et al., 2001). Several theories have been put forth to account for this behavior, most recently by Daniel and Danson (2010), Arcus and coworkers (Hobbs et al., 2013; Arcus et al., 2016), and Warshel and coworkers (Roy et al., 2017). Deviations from typical Eyring behavior are generally ascribed to heat capacity (*C*_*p*_) effects, specifically the change in heat capacity upon ligand binding during the catalytic transition state ($\Delta C_{p}^{†}$).

Moreover, it is clear from Figure 2 that ln(*k*_*cat*_) is non-linear with temperature for the basal reaction, but shows a linear Eyring plot for PRFAR-activated IGPS. The observed curvature in the basal reaction indicates that Δ*G*^†^ (and thus Δ*H*^†^ and Δ*S*^†^) are not temperature independent, meaning$\Delta C_{p}^{†}\neq 0$. The plot in Figure 2C yields Δ*C*_*p*_ = −2.9 ± 0.6 kJ/mol-K. This value is similar to the negative heat capacity change observed in the interaction between methylthioadenosine phosphorylase and its transition state analog (Guan et al., 2011), as well as transcription factor/DNA binding (Bergqvist et al., 2004). The negative Δ*C*_*p*_ observed for basal Gln hydrolysis is similar to, but slightly less than, values reported for other enzymes (Hobbs et al., 2013). Non-linear plots of reaction rate vs. temperature have also been suggested to result from denaturation, aggregation, or rapid folding/unfolding of the enzyme. However, several control experiments (see section Materials and Methods) indicate this is not the case with IGPS. In addition, the onset of curvature in the Eyring plot for the basal IGPS reaction occurs well-below the denaturation temperature of IGPS. The negative heat capacity for the basal reaction likely originates from decreases in vibrational and rotational modes and/or changes in hydration (Eisenberg and Crothers, 1979; Gomez et al., 1995). The strikingly different temperature dependencies for the Gln hydrolysis reactions suggest that PRFAR, in part, accelerates the hydrolytic reaction by altering critical aspects of the chemical mechanism that are fundamentally different than the basal reaction.

It is also evident that PRFAR is a much weaker allosteric activator at the growth temperature of *T. maritima* than at the more conveniently studied room temperature (Figure 2). The difference in free energy (Δ*G*^†^) for Gln hydrolysis between PRFAR-activated and apo IGPS decreases from 17.6 to 9 kJ/mol between 303 and 343 K. At the growth temperature of *T. maritima*, the energetic effect of PRFAR over the basal hydrolytic rate is attenuated by nearly 9 kJ/mol primarily due to the increase in basal rates relative to that of the PRFAR-activated enzyme. It is interesting that basal activity is very similar at temperatures ≥323 K, which may be explained in part by a large growth temperature window of this organism. Although the ideal environment for *T. maritima* is a water temperature of ~ 353 K, it is known to grow over a range of 328–363 K (Huber et al., 1986).

In addition, the large temperature range studied allows for a more robust examination of the effect of PRFAR on the *K*_*m*_-value for Gln. It is clear from Figure 2 and Table 1 that the *K*_*m*_ for Gln is ~3-fold weaker in the absence of PRFAR, indicating that in addition to its V-type allostery, PRFAR also exhibits weak K-type allostery as well (Figure 2D). Like the *k*_*cat*_-values determined from kinetic traces, the Gln *K*_*m*_-values are independent of temperature in the presence of PRFAR, but decrease by 1.6-fold between 303 and 353 K in studies of basal activity. Thus, Gln has higher steady-state affinity for IGPS at 353 K than it does at room temperature, and a van't Hoff analysis (Figure 2) yields a free energy of Gln binding of −3.6 ± 0.2 kJ/mol. The apparent coupling free energy (*Q*_*ax*_) between PRFAR and Gln was obtained at each temperature from Equation (7). From Equation (8) the apparent coupling entropy and enthalpy were both negative (Figure S8). Negative coupling entropy values have been observed in other thermophilic organisms (Tlapak-Simmons and Reinhart, 1998; McGresham et al., 2014).

At room temperature, previous studies suggested an important role for ms motions in the allosteric activation of IGPS by PRFAR (Amaro et al., 2007; Lipchock and Loria, 2009, 2010; Rivalta et al., 2012, 2016; Manley et al., 2013; Lisi et al., 2016, 2017). Here, we extended these studies to examine the temperature dependence of conformational flexibility in apo and PRFAR-bound IGPS at 303, 322, and 343 K. Apo IGPS dynamics are enhanced significantly at temperatures above 323 K, and the number of ILV methyl groups with millisecond timescale flexibility is nearly identical to that of PRFAR-bound IGPS, even in the absence of the PRFAR activator, at 343 K. For residues with measurable dispersion curves, the exchange rate constants, *k*_*ex*_, were examined with Arrhenius plots (Figure 6) to determine apparent activation energies for these motional processes. These values are plotted along the IGPS primary sequence in Figure 6C and mapped onto the structure in Figure 6D. In general, the activation barriers to motion are higher for residues in the apo enzyme (Mean ± St. Dev.; 18.5 ± 14.0 kJ/mol) than those of the PRFAR bound enzyme (12.9 ± 13.6 kJ/mol), with two exceptions, L50 and L169. There is not an obvious reason for the different activation barriers, as residues that are completely buried or completely exposed to solvent have similar *E*_*a*_-values, nor is there a correlation between *E*_*a*_ and secondary structure elements. Overall, the close link between ms motions and allosteric activation is not as evident near the elevated growth temperatures of *T. maritima*. Therefore, it would seem that the mechanism of PRFAR allostery is somewhat different at 343 K than it is at 300 K and not clearly linked to ms motions.

A community network analysis that was previously used to understand allosteric pathways in IGPS was further examined here to aid in explaining the different activation barriers for conformational motion (Rivalta et al., 2012). Residues from our NMR analysis that fall in the same communities designated by Rivalta and coworkers have similar activation energies, albeit with some exceptions. For example, the majority of residues available for Arrhenius analysis in apo IGPS correspond to those in communities F2 and F3. Residues in community F3 have *E*_*a*_-values clustered around the community average (16 kJ/mol), with the exception of I6, L50, and I93, all of which have *E*_*a*_ > 30 kJ/mol. The disagreement between these values with the rest of those in community F3 suggests these residues may switch dynamic communities upon PRFAR binding (Rivalta et al., 2012; Lisi et al., 2017). Similarly, the motional activation energy of V157 is significantly higher than all other residues in community F2, the other community encompassing the majority of our available NMR data on apo IGPS. Correlation of this previous network analysis to Arrhenius values determined from NMR studies on PRFAR-bound IGPS also reveal that residues within the dominant communities (F2' and F3') of this binary complex have relatively similar activation energies. We also extended these correlations to include temperature-dependent chemical shifts presented in Figure 3. Residues that fall outside linearity in the carbon and proton dimensions overwhelmingly belong to communities F2 and F3 as well, suggesting that these clusters most strongly influence structural and dynamic activation of IGPS over a wide temperature range. These data suggest that residues may switch network communities in a temperature dependent manner.

The data presented herein highlight a complex temperature dependence that affects the conformational flexibility and catalytic activity of IGPS. Most interestingly, temperatures more closely approximating those of the native *T. maritima* environment drastically enhance ms dynamics in both apo and PRFAR-bound IGPS. However, the similar levels of conformational flexibility observed by NMR in apo and PRFAR-bound IGPS at 343 K does not translate to equal catalytic activities, indicating that there must be other effects elicited by PRFAR binding other than enhancement of ms motions that enable efficient Gln hydrolysis. These temperature dependent studies indicate an additional layer of complexity in the allosteric activation of IGPS since numerous kinetic and thermodynamic parameters for this enzyme show differing and varying response to elevated temperatures.

## Author contributions

GL: Designed and performed the research, analyzed the data, and wrote the paper; AC: Performed the research; JPL: Designed the research, analyzed the data, and wrote the paper.

### Conflict of interest statement

The authors declare that the research was conducted in the absence of any commercial or financial relationships that could be construed as a potential conflict of interest.
