# Supplementary material for: Glutamine Hydrolysis by Imidazole Glycerol Phosphate Synthase Displays Temperature Dependent Allosteric Activation
Source: Front Mol Biosci. 2018 Feb 6;5:4. doi: 10.3389/fmolb.2018.00004 (PMC5808140; doi:10.3389/fmolb.2018.00004)
Supplement: Supplementary file 1 [file DataSheet1.DOCX]

Supplementary Material

**Glutamine hydrolysis by imidazole glycerol phosphate synthase displays temperature dependent allosteric activation**

**George P. Lisi, Allen A. Currier, and J. Patrick Loria***

*** Correspondence:** J. Patrick Loria: patrick.loria@yale.edu

**Tables S1-S4.** NMR summary of Ile, Leu, and Val methyl groups undergoing conformational exchange in apo- and PRFAR-bound IGPS at 30, 50, and 70 °C and related conformational exchange parameters

**Figure S1.** Far-UV circular dichroism measurements of apo IGPS at 30, 50, and 70 °C

**Figure S2.** Temperature dependent ^1^H NMR spectra of glutamine

**Figure S3.** Determination of k_Act_ for PRFAR and Arrhenius plots based on glutaminase kinetic assays

**Figure S4.** ^13^CH_3_-ILV methyl NMR spectral overlays of apo and PRFAR-bound IGPS at 30, 50, and 70 °C

**Figure S5.** ^1^H^15^N NMR spectral titration of IGPS with PRFAR and temperature dependence of PRFAR ligand

**Figure S6.** Temperature dependent proton and carbon chemical shifts of apo and PRFAR-bound IGPS

**Figure S7.** Summary of structural flexibility and histogram distributions of k_ex_ values determined from NMR relaxation experiments for apo- and PRFAR-bound IGPS at 30, 50, and 70 °C

**Supplementary Table 1.** Temperature dependence of Ile, Leu, and Val methyl groups undergoing conformational exchange in apo (top) and PRFAR-bound (bottom) IGPS. Residues correspond to ^2^H,^13^CH_3_-ILV,^15^N labeled HisF.

**Supplementary Table 2.** Conformational exchange parameters determined from ^13^CH_3_-ILV relaxation dispersion analysis of apo (gray) and PRFAR-bound (purple) IGPS at 30 °C.

**Supplementary Table 3.** Conformational exchange parameters determined from ^13^CH_3_-ILV relaxation dispersion analysis of apo (gray) and PRFAR-bound (purple) IGPS at 50 °C.

**Supplementary Table 4.** Conformational exchange parameters determined from ^13^CH_3_-ILV relaxation dispersion analysis of apo (gray) and PRFAR-bound (purple) IGPS at 70 °C.


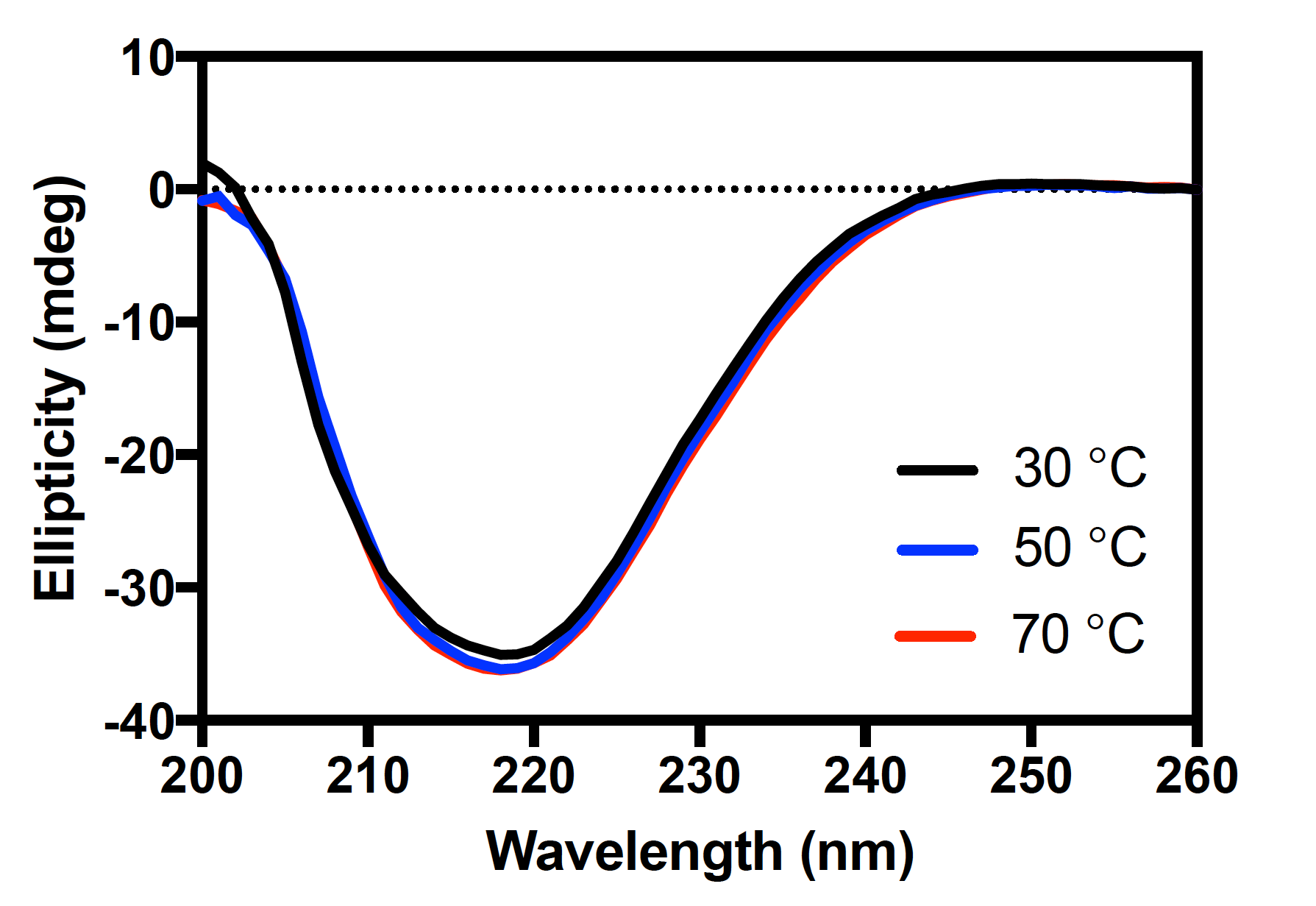


**Supplementary Figure 1.** Far-UV circular dichroism traces of IGPS following 2 hour equilibration at 30 (black), 50 (blue), or 70 °C (red). Each trace was signaled averaged over four scans.


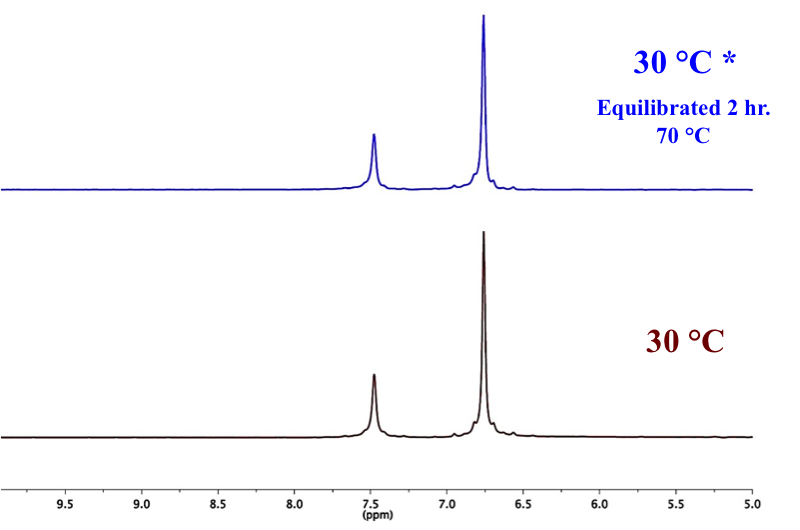


**Supplementary Figure 2.** Temperature dependent ^1^H NMR spectra of ^15^N-labeled Gln. Identical ^1^H NMR spectra of ^15^N Gln collected at 30 °C before (maroon) and after a two hour equilibration at 70 °C (blue). Following equilibration, 88 % of the peak intensity remains.


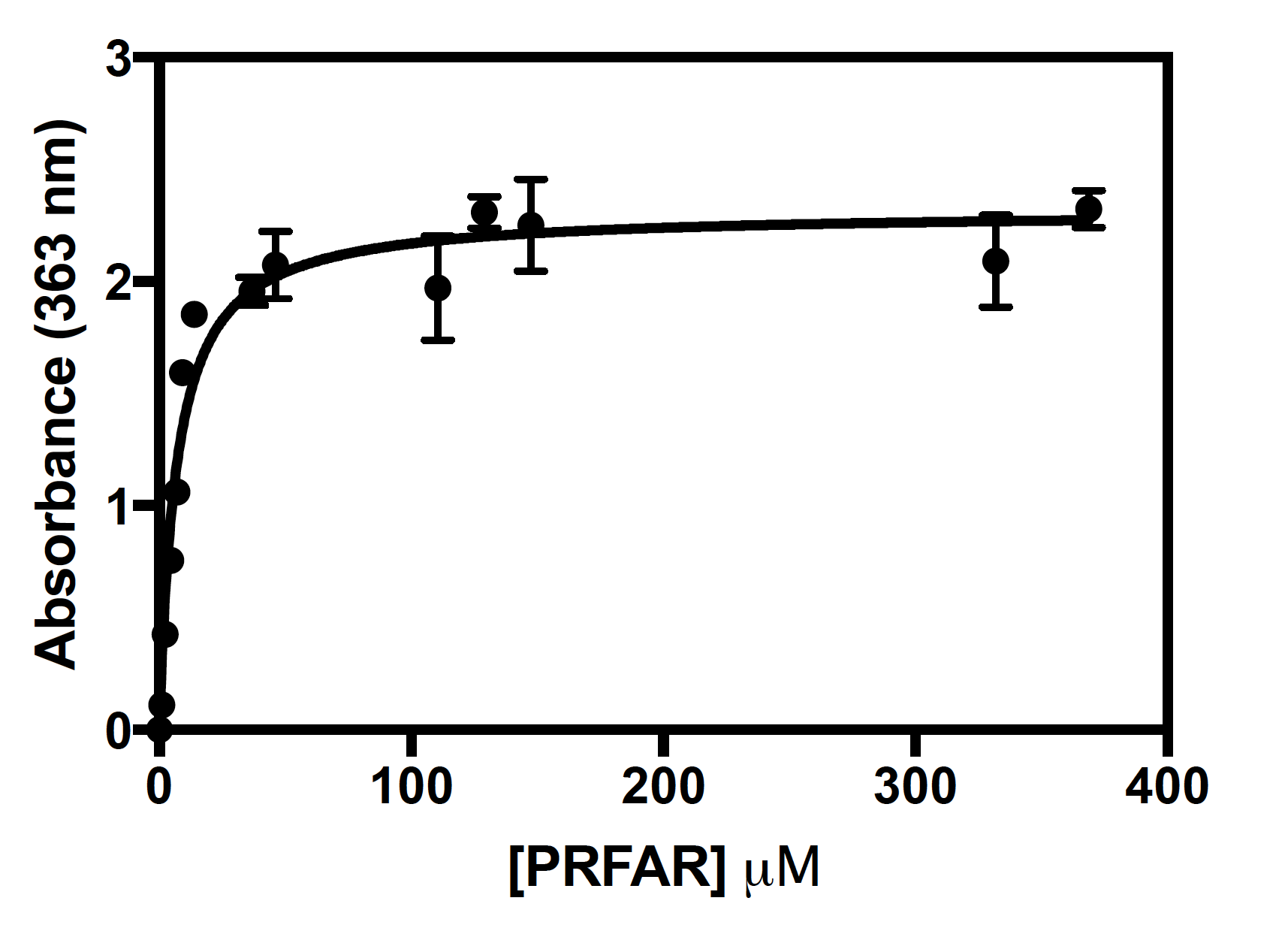


**Supplementary Figure 3.** Determination of *K*_Act_ from PRFAR-dependent glutaminase assays. The concentration of glutamine was held constant (14 mM) and the concentration of PRFAR was varied, yielding K_Act_ (PRFAR) = 6.6 μM.


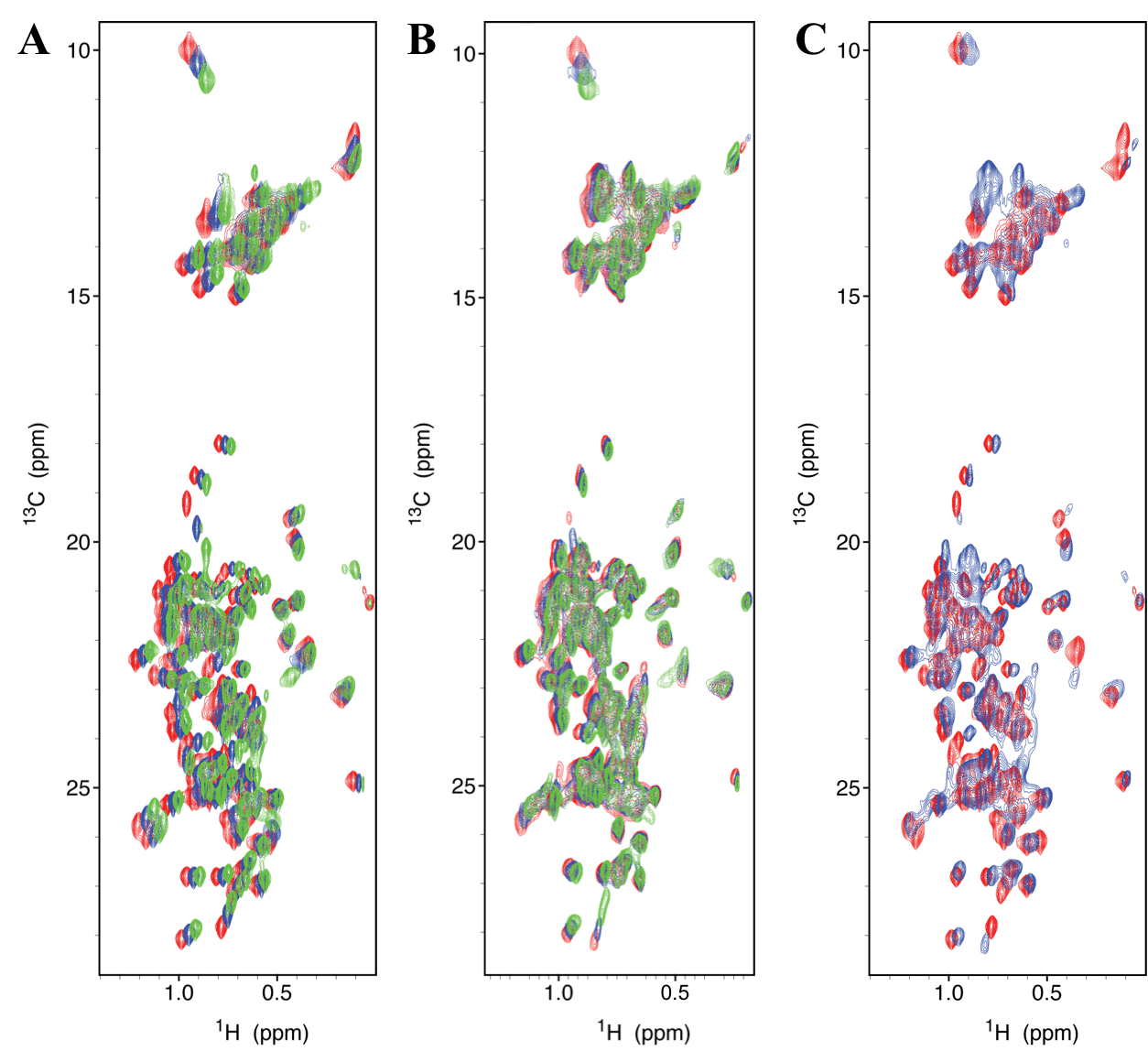


**Supplementary Figure 4. (A)** Overlays of ^13^CH_3_ ILV NMR spectra of apo IGPS collected at 30 °C (red), 50 °C (blue), and 70 °C (green). **(B)** Overlays of ^13^CH_3_ ILV NMR spectra of PRFAR-bound IGPS collected at 30 °C (red), 50 °C (blue), and 70 °C (green). **(C)** ^13^CH_3_ ILV spectral comparison of apo IGPS (red) and PRFAR-bound IGPS (blue) at 30 °C.


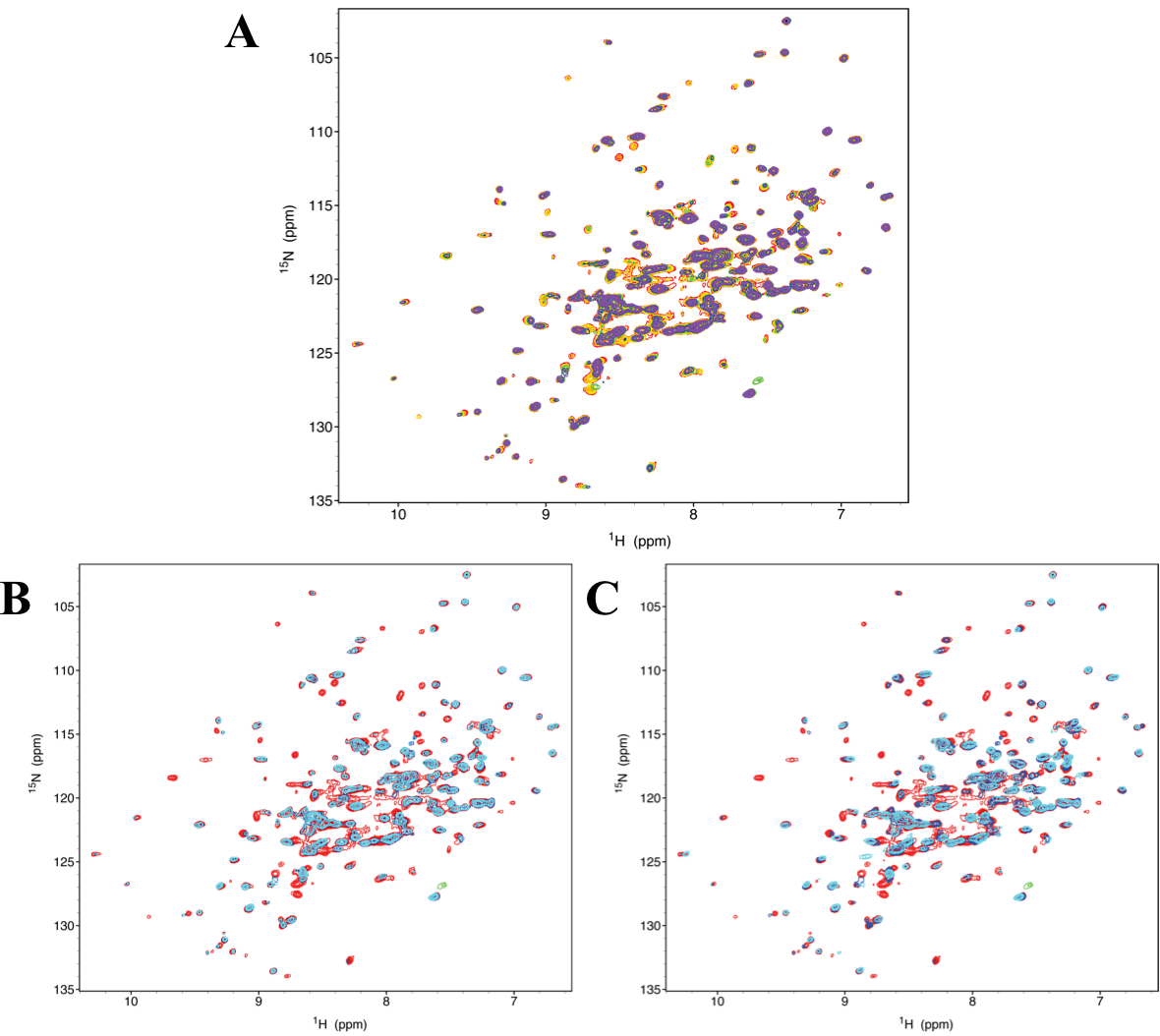


**Supplementary Figure 5. (A)** NMR titration of apo IGPS with PRFAR monitored by ^1^H^15^N HSQC spectral overlays ([PRFAR] 0 – 1.1 mM, red 🡪 purple). **(B)** Test of the stability of the PRFAR ligand at high temperatures. NMR spectra of apo IGPS (red) and PRFAR saturated IGPS (blue) collected at 30 °C overlaid with a spectrum of PRFAR-bound IGPS from a sample equilibrated at 70 °C for ~ 3 hours (cyan). **(C)** Identical NMR spectra of apo IGPS (red) and PRFAR saturated IGPS (blue) shown in **B**, now overlaid with a spectrum of PRFAR-bound IGPS from a sample equilibrated at 70 °C for ~ 80 hours (cyan).


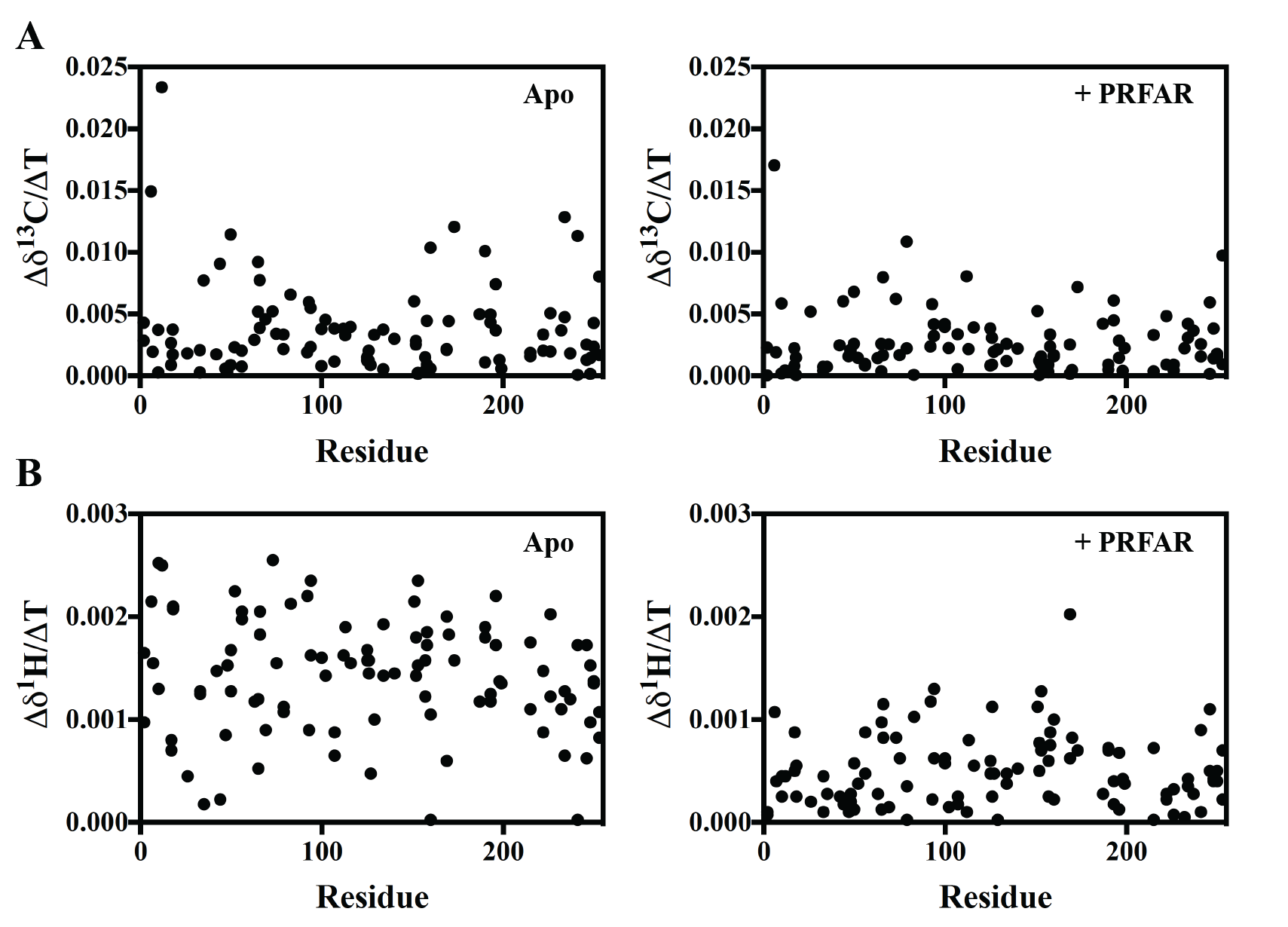


**Supplementary Figure 6.** Temperature dependence of IGPS chemical shifts in ^1^H^13^CH_3_-ILV NMR spectra. **(A)** Differences in carbon chemical shifts (Δδ^13^C) divided by the difference between temperatures (40 °C) for each ILV resonance in IGPS. **(B)** Differences in proton chemical shifts (Δδ^1^H) divided by the difference between temperatures (40 °C) for each ILV resonance in IGPS. **Note:** the magnitudes of all shifts are normalized to be positive values, and in many cases, multiple points exist for the same residue, corresponding to the δ^1^ and δ^2^ methyl groups of leucine or the γ^1^ and γ^2^ methyl groups of valine.


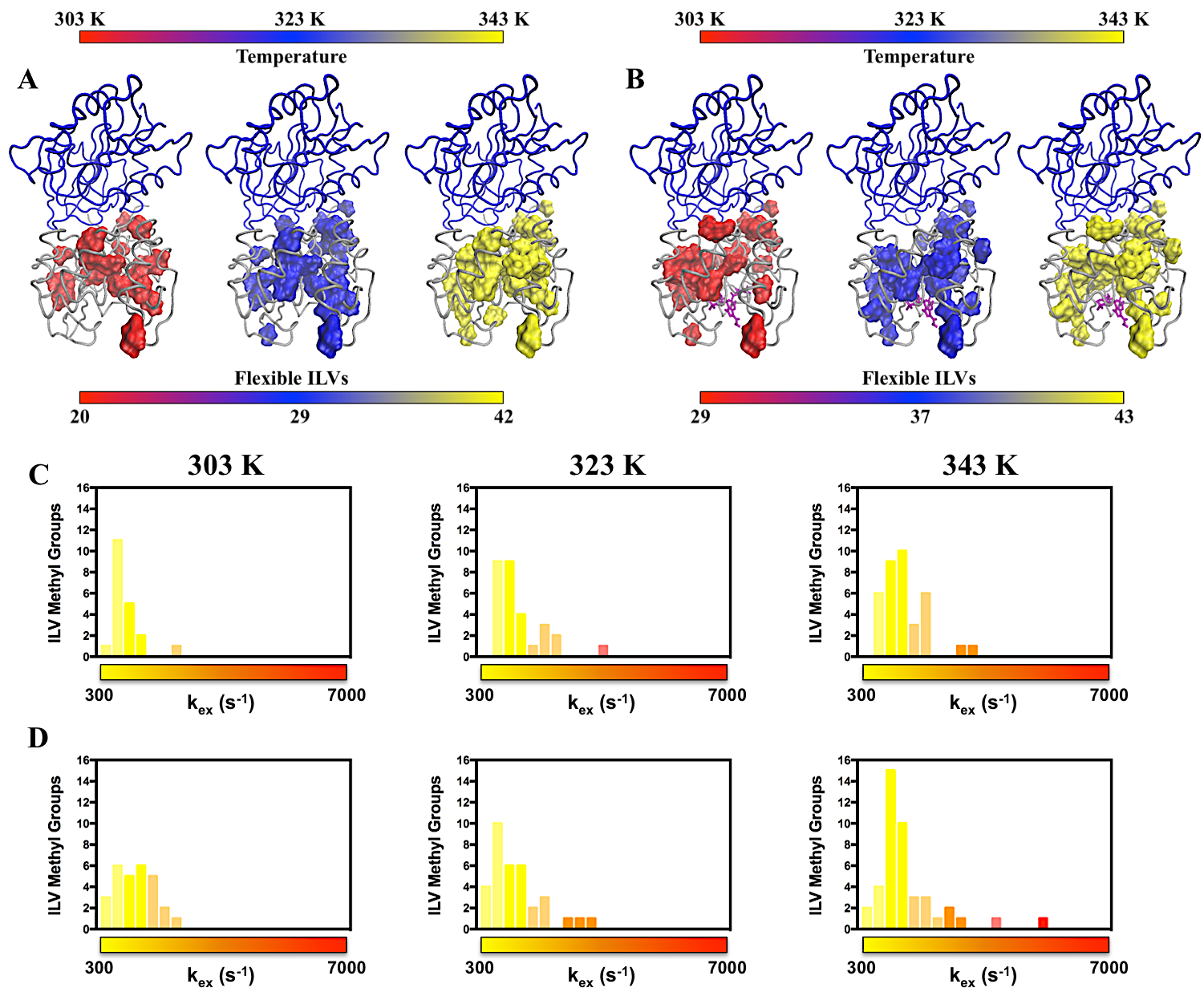


**Supplementary Figure 7.** Flexible ILV residues in **(A)** apo and **(B)** PRFAR-bound IGPS determined from relaxation dispersion NMR experiments at 303 K (red), 323 K (blue), and 343 K (yellow). The PRFAR ligand is shown in purple sticks in **(B)**. Temperatures are indicated by the top gradient and flexible ILV residues corresponding to those points are shown by the bottom gradients. Histograms showing clustering of k_ex_ values determined from relaxation dispersion NMR experiments for **(C)** apo and **(D)** PRFAR-bound IGPS at 30, 50, and 70 °C. Optimal bin sizing for these histograms was determined using a procedure outlined in Scott, D.W. *Biometrika*. **1979**. *66*. 605-610.


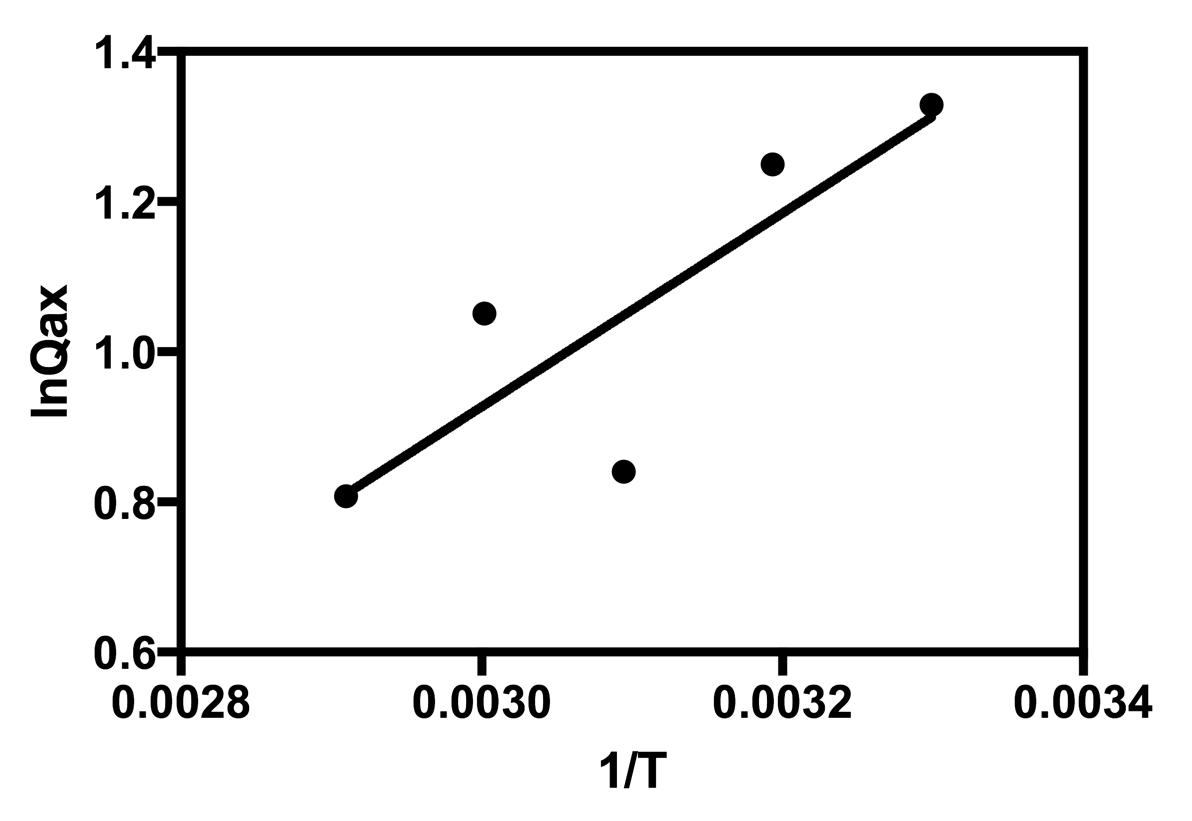


**Supplementary Figure 8.** Apparent coupling thermodynamics. The apparent coupling free energy between PRFAR and Gln, are obtained from temperature dependent Gln K_m_ values and determined from equation 7 in the main text. The solid line is a fit of equation 8 (main text) to the data points. The resulting apparent coupling entropy and enthalpy are –24.4 ± 12 J/mol-K and –10.7 ± 4.0 kJ/mol, respectively.
